# Preoperative stiffness is the most important predictor of postoperative patient’s satisfaction after total knee arthroplasty

**DOI:** 10.1007/s00590-023-03526-w

**Published:** 2023-03-22

**Authors:** Mohammed Anter Abdelhameed, Mohammad Kamal Abdelnasser, Bishoy Raafat Zaky, Hatem M. Bakr, Mirette Aziz, Mohamed Mahran

**Affiliations:** 1 Orthopaedic and Traumatology Department, Assiut University Hospital, Assiut, Egypt; 2 Public health department, Faculty of Medicine, Assiut University, Assiut, Egypt

**Keywords:** Total knee arthroplasty, Patient-reported outcome measures, Stiffness

## Abstract

**Purpose:**

To predict the most important preoperative factor affecting the patient satisfaction after total knee arthroplasty (TKA) in trial to improve patient counselling process.

**Methods:**

We retrospectively reviewed all patients who underwent primary TKA from January 1, 2018, to January 31, 2019, with minimum one-year follow-up for the previously collected patient-reported outcome measures (PROMs) as Oxford Knee Score (OKS) and Knee Injury and Osteoarthritis Outcome Score for joint replacement (KOOS, JR) preoperative, 6 months and 12 months postoperative.

**Results:**

By using Oxford knee score at 12 months as dependent variable, we found a negative moderate spearman correlation between age and Oxford knee score at 12 months postoperative. Moderate negative spearman correlation was also found between Oxford knee score at 12 months postoperative and KOOS pain, stiffness and function scores at preoperative and 6 months postoperative, denoting higher satisfaction at 12 months with less perceived stiffness, pain and limited function at baseline and 6 months postoperative. A multivariate regression analysis was done using the oxford knee score at 12 months as dependent variable to detect the predictors of oxford knee score at 12 months postoperatively and showed that younger age and less perception of stiffness at baseline were significant predictors of higher Oxford knee score: higher satisfaction at 12 months postoperative.

**Conclusion:**

Preoperative stiffness can predict the postoperative satisfaction score more than any other factor. We also address the importance of combining more than one PROM in assessing patients as OKS and KOOS, JR.

## Introduction

Despite being one of the most successful procedures in the last fifty years, total knee arthroplasty (TKA) still has a considerable postoperative dissatisfaction rate reaching 15–20% in some studies [1–4]. The outcomes of TKA can be assessed with various methods: implant survivorship, radiological assessment, clinical assessment and patient-reported outcome measures (PROMs) [5]. While the first three modalities of assessment are objective, PROMs can provide a subjective measure of the patients’ perception of the success of an intervention [6]. Improving patient’s quality of life and achieving patient satisfaction are now considered the major goal of joint replacement surgeries. Consequently, the outcome assessment scores have been shifted to the use of PROMs. Moreover, disease-specific measures such as Oxford Knee Score (OKS) [7] and Knee Injury and Osteoarthritis Outcome Score for joint replacement (KOOS, JR) [8] are now widely used for predicting the outcome after knee replacement surgery with preoperative pain and functional status, as measured by PROMs, and are used to predict pain and functional ability after TKA [6, 9, 10]. Interestingly, though patients with higher levels of preoperative pain and disability demonstrate the greatest improvements in PROM scores, they do not achieve absolute postoperative scores comparable to patients with less preoperative pain and better baseline function [11, 12]. Indeed, patient satisfaction is a multifactorial issue that is related to many factors other than the surgery itself including operative factors (type of surgery, type of anaesthesia, operative time, complications, implant type, etc.), postoperative factors (postoperative care, analgesics and pain management, hospital experience, etc.) and preoperative factors [13]. The purpose of this study is to predict the most important preoperative factor affecting the patient satisfaction after TKA through correlating the OKS, which is the most widely used and interpreted score, with another more detailed score (KOOS, JR) in which stiffness, function and pain are separate items and can be separately interpreted in an attempt to improve patient counselling process before TKA.

## Materials and methods

After IRB approval, we retrospectively reviewed the prospectively collected data of all patients who underwent primary TKA for knee OA in the period from July 1, 2018, to July 1, 2019, with a minimum of one-year follow-up. Only patients who had unilateral primary TKA for OA were included. Revision TKA or those with inflammatory arthritis previous knee surgery were excluded. Also, patients who did not complete their PROM scores were excluded. One hundred patients met our inclusion criteria and were reviewed for their preoperative, 6 months and 12 months postoperative PROM scores (OKS and KOOS, JR). Preoperative demographic data such as age, sex, marital status and smoking, and comorbidities such as hypertension and diabetes mellitus (DM) and previous operations were also collected.

### Statistical analysis

Data were entered and analysed using SPSS (SPSS Statistics for Windows, version 27.0, NY). Descriptive analysis was performed for the participants’ socio-demographic data. We used Friedman’s ANOVA test for repeated measures analysis of the Oxford knee scores at baseline, 6 months and 12 months postoperative to determine whether there was a significant improvement in the measured scores with time. We also used Wilcoxon signed-rank test (with Bonferroni correction) as a post hoc test to detect the significant differences between each two points of time of the repeated assessments. Spearman correlation was used to test the association between quantitative factors that could be associated with total Oxford knee score at 12 months postoperative such as age, scores of stiffness, function and pain at baseline, 6 and 12 months postoperative as measures by KOOS, JR. Mann–Whitney U test was used to detect the association between total Oxford knee score and sex of patients, having a comorbidity, such as diabetes or hypertension, and having a previous operation. Multivariate regression was performed to identify the predictors of higher total Oxford knee score at 12 months postoperative. Significant variables in bivariate analysis were entered in the regression model. We adjusted for age, sex and preoperative KOOS scores by adding these variables to the model. *P* value of < 0.05 was used as the level of significance for all the statistical tests.

## Results

The mean age of the collected sample was 59. 73 ± 7.15 (range 35–75). Table 1 shows the basic demographic data of the collected sample.

**Table 1:** Characteristics of the study sample

Age (mean ± SD) (Range)	59.73 ± 7.15 (35–75)
Sex	
Male	23 (23.0)
Female	77 (77.0)
Diagnosis	
Osteoarthritis	87 (87.0)
Bilateral osteoarthritis	8 (8.0)
Rheumatoid arthritis	5 (5.0)
Previous operation	
Yes	29 (29.0)
No	70 (70.0)
Comorbidities	
Hypertension	
Yes	25 (25.0)
No	75 (75.0)
Diabetes	
Yes	15 (15.0)
No	85 (85.0)

Repeated measures of preoperative, 6 and 12 months postoperative of oxford and KOOS, JR scores showed a significant differences in the ranked scores of OKS total score, KOOS, JR pain, KOOS, JR function and KOOS, JR stiffness dimensions of KOOS, JR scale at the three time points of the study: preoperative, 6 months and 12 months postoperative, with the best observed scores at 12 months postoperative, reflecting that the highest patients' satisfaction is obtained at this point (Table 2).

**Table 2:** Repeated measures of baseline, 6 and 12 months postoperative scores of oxford and KOOS scales

	Baseline	6 month postoperative	12 months postoperative
Oxford total score			
Median (interquartile range)	17.0 (12.0–24.0)	34.0 (25.0–0.34)	43.0 (37.0–0.43)
*χ*^2^	168.36		
*P* value	< 0.001		
KOOS stiffness			
Median (interquartile range)	3.0 (3.0–4.0)	2.0 (1.0–2.0)	0.0 (0.0–1.0)
*χ*^2^	198.53		
*P* value	< 0.001		
KOOS pain			
Median (interquartile range)	14.0 (13.0–15.0)	8.0 (7.0–10.0)	2.0 (1.0–3.0)
*χ*^2^	200.00		
*P* value	< 0.001		
KOOS function			
Median (interquartile range)	8.0 (7.0–8.0)	4.0 (4.0–0.6)	1.5 (1.0–2.0)
*χ*^2^	200.00		
*P* value	< 0.001		

Table 2 shows the results of Friedman’s ANOVA test for repeated measures analysis. The table shows that we have significant differences in the median of scores of oxford scale, KOOS pain, KOOS function and KOOS stiffness dimensions of KOOS scale at the three time points of the study: baseline, 6 months and 12 months postoperative, with the best observed scores, reflecting higher patients satisfaction at 12 months postoperative

*Friedman’s ANOVA

We also compared the paired differences in OKS and KOOS, JR scores between preoperative, 6 months postoperative and 12 months postoperative, and a significant difference was found between each of the pairs (P < 0.016) as measured by Wilcoxon signed-rank test (with Bonferroni correction). This shows that OKS total score significantly improved between preoperative and 6 months postoperative (*z* = − 7.50, *P* =  < 0.001), between 6 and 12 months postoperative (*z* = − 8.73, *P* =  < 0.001), and also between preoperative and 12 months postoperative (*z* = − 8.66, *P* =  < 0.001), denoting that the best improvement was in the first 6 months postoperatively (*Z* = − 7.50) with continued improvement from 6 to 12 months. Significant improvement in KOOS, JR stiffness, pain and function scores was also observed (Table 3).

**Table 3:** Paired differences in oxford and KOOS scores between baseline, 6 months postoperative and 12 months postoperative

	6 months postoperative- baseline	12 months postoperative- baseline	12 months postoperative- 6 months postoperative
Total Oxford knee score			
*Z*	− 7.50	− 8.66	− 8.73
*P* value	< 0.001	< 0.001	< 0.001
KOOS stiffness			
*Z*	− 8.88	− 9.10	− 8.92
*P* value	< 0.001	< 0.001	< 0.001
KOOS pain			
*Z*	− 8.72	− 8.72	− 8.79
*P* value	< 0.001	< 0.001	< 0.001
KOOS function			
*Z*	− 8.81	− 8.77	− 8.83
*P* value	< 0.001	< 0.001	< 0.001

Table 3 shows that there is a significant difference (with Bonferroni correction) between each of the pairs (*P* < 0.016). The table shows that oxford total score significantly improved between baseline and 6 months postoperative (*z* =  − 7.50, *P* =  < 0.001), between baseline and 12 months postoperative (*z* =  − 8.66, *P* =  < 0.001) and between 6 and 12 months postoperative (*z* =  − 8.73, *P* =  < 0.001). Significant improvement in KOOS stiffness, pain and function scores was also observed

*Wilcoxon signed-rank

By using OKS at 12 months as dependent variable indicating patient satisfaction, we correlated between OKS total score at 12 months postoperative, age and KOOS, JR (stiffness, pain and function scores) at preoperative, 6 and 12 months postoperative and we found that there was a negative moderate spearman correlation between age and OKS score at 12 months postoperative: The younger the age, the higher the score. Moderate negative spearman correlation was also found between OKS score at 12 months postoperative and KOOS, JR pain, stiffness and function scores at preoperative and 6 months postoperative, denoting higher satisfaction at 12 months with less perceived stiffness, pain and limited function at baseline and 6 months postoperative. Moreover, strong negative correlation was found between KOOS, JR stiffness and function at 12 months postoperative and OKS score at 12 months postoperative. All observed correlations were statistically significant (Table 4).

**Table 4:** Correlation between oxford total score at 12 months postoperative, age and KOOS scores at baseline, 6 and 12 months postoperative

	Oxford knee score—12 m postoperative (r value)	*P* value
Age	− 0.668	< 0.001
*KOOS baseline*		
Stiffness	− 0.565	< 0.001
Function	− 0.514	< 0.001
Pain	− 0.451	< 0.001
*KOOS 6 months postoperative*		
Stiffness	− 0.590	< 0.001
Function	− 0.623	< 0.001
Pain	− 0.542	< 0.001
*KOOS 12 months postoperative*		
Stiffness	− 0.839	< 0.001
Function	− 0.763	< 0.001
Pain	− 0.625	< 0.001

Table 4 shows that there was a negative moderate spearman correlation between age and Oxford knee score at 12 months postoperative: The younger the age, the higher the score. Moderate negative spearman correlation were also found between Oxford knee score at 12 months postoperative and KOOS pain, stiffness and function scores at baseline and 6 months postoperative, denoting higher satisfaction at 12 months with less perceived stiffness, pain and limited function at baseline and 6 months postoperative. Moreover, strong negative correlation was found between KOOS stiffness and function at 12 months postoperative and Oxford knee score at 12 months postoperative. All observed correlations were statistically significant

*Spearman correlation

We also assessed the association between OKS total score at 12 months postoperative as dependent variable and sex, comorbidities and previous operations, and found that having comorbidity such as hypertension or diabetes was associated with lower OKS scores at 12 months postoperative. Other tested variables were found to be not significantly associated with Oxford knee score, such as sex and having a history of previous surgery (Table 5).

**Table 5:** Association between oxford total score at 12 months postoperative and sex, comorbidities and previous operations

	Oxford knee score—12 m postoperative^*^ Median (interquartile range)	*P* value
Sex		
Male	43.0 (36.0–0.43)	0.307
Female	43.0 (37.0–44.0)	
Hypertension		
Yes	41.0 (34.5–0.43)	0.019
No	43.0 (39.0–44.0)	
Diabetes		
Yes	36.0 (36.0–43.0)	0.010
No	43.0 m (39.5–43.5)	
Previous operation		
Yes	43.0 (36.0–43.0)	0.326
No	43.0 (37.0–44.0)	

Table 5 shows that having comorbidity such as hypertension or diabetes was associated with lower Oxford knee scores at 12 months postoperative. Other tested variables were found to be not significantly associated with Oxford knee score, such as sex and having a history of previous surgery

*Mann–Whitney *U* test

A multivariate regression analysis was done using the OKS at 12 months as dependent variable to detect the predictors of oxford knee score at 12 months postoperatively and this linear regression showed that younger age and less perception of stiffness at baseline were significant predictors of higher Oxford knee score: higher satisfaction at 12 months postoperative (Table 6).

**Table 6:** Predictors of Oxford knee score at 12 months postoperative

	Unstandardized B	Standard error	*P* value
Age	− .260	.049	0.000
Hypertension	1.297	.727	.078
DM	1.360	.895	.132
KOOS stiffness baseline	− 2.784	.692	0.000
KOOS pain total baseline	− .501	.392	.204
KOOS function total baseline	.168	.763	.826

Table 6 shows the results of linear regression. It shows that younger age and less perception of stiffness at baseline were significant predictors of higher Oxford knee score: higher satisfaction at 12 months postoperative

## Discussion

The most important finding of this study is that age and preoperative stiffness are the most important predictors of patient satisfaction after TKA. Moreover, PROMs continue to improve till one year postoperatively. This study has also showed that satisfaction after TKA can be predicted preoperatively through the preoperative patient perception of stiffness which greatly affects patient satisfaction scores postoperatively. These findings have special benefits in improving patients' counselling prior to surgery in terms of patient’s expectations of the degree of postoperative improvement and time of maximum improvement.

It is already well known in the literature that patients with better preoperative pain and functional status achieve better postoperative pain and function [6, 9, 14–16] and that when using the change in score (difference in pre- and postoperative score) as the outcome, those with worse pain and function scores get the greatest improvement, but never return to the same level of function as those with the least preoperative pain and functional limitation [14, 17, 18]. However, in this study we concluded that preoperative stiffness is more important to affect postoperative patient outcome rather than pain which, to our knowledge, have not been mentioned before. In addition, we correlated the OKS, which is the most widely used and interpreted score, with another more detailed score (KOOS, JR) in which the items of stiffness and pain are separate and allow a more detailed interpretation.

Goh et al. [19] have used OKS and knee society score (KSS) which were collected retrospectively as predictor for patient satisfaction 2 years after TKA and concluded that early postoperative scores, specifically the OKS and KSS, can predict patient satisfaction at 2 years after TKA with good accuracy. Canfield et al. [20] stated that there is no need to measure PROMs after 6 months as there is little improvement. Both finding were consistent with our study, as despite continued significant improvement in OKS and KOOS, JR till one year postoperatively was observed, and the improvement in PROMs between 6 and 12 months was little compared to the great improvement in the first six months. However, we suggest that PROMs are best to be measured at 12 months after TKA to be more informative and to actually represent the highest level of patient satisfaction.

Remarkably, there is a great discrepancy in the literature assessing the satisfaction after TKA with the authors reporting satisfaction rates ranging from 75 [21] to 97% [22]. This variation in satisfaction rates may in part be related to the way in which satisfaction is currently assessed, using various questions with diverse responses, and different combinations of responses to define satisfaction. This study has shed the light on the importance of using a common validated way to assess patient's satisfaction as we address the importance of combining more than one PROM score in assessing patients' satisfaction as (OKS and KOOS, JR). This is because the stiffness component of KOOS, JR is not present in OKS; moreover, questions of pain and function in OKS cannot be separately interpreted as in KOOS, JR.

This study has demonstrated that less stiffness and better range of motion (ROM) preoperatively are accompanied by better OKS and better patient satisfaction postoperatively. Also, younger patients experienced better outcome. This is partly explained as younger patients would have better ROM preoperatively and less comorbidities. Clement et al. [23] concluded that age of less than 55 years is not an independent predictor of functional outcome or rate of patient satisfaction after TKA which is consistent with our finding as age is not an independent factor affecting patient satisfaction rather than an associated factor with stiffness.

### Strengths and limitations

The small sample size of the study and its retrospective nature were the main limitations. However, the added strength of using 2 PROM scores which improved the analysis of the components of patient satisfaction and the improved counselling of patients that the maximum improvement will be in the first 6 months postoperatively and it is related to their preoperative ROM are good points.

## Conclusion

Preoperative stiffness can affect the postoperative satisfaction score more than any other factor. We also address the importance of combining more than one PROMs in assessing patient's satisfaction as OKS and KOOS, JR.

## Data Availability

All data generated or analysed during this study are included in this published article.
